# Supplementary material for: The Identification and Cytotoxic Evaluation of Nutmeg (Myristica fragrans Houtt.) and Its Substituents
Source: Foods. 2023 Nov 22;12(23):4211. doi: 10.3390/foods12234211 (PMC10706638; doi:10.3390/foods12234211)
Supplement: Supplementary file 1 [file foods-12-04211-s001.zip › foods-2671076-supplementary.pdf]

Identification and Cytotoxic Evaluation of Nutmeg (*Myristica fragrans* Houtt.) and Its Adulterants

Suthiwat Khamnuan [1], Ampai Phrutivorapongkul [1], Pornsiri Pitchakarn [2], Pensiri Buacheen [2], Jirarat Karinchai [2], Chuda Chittasupho [1], Mingkwan Na Takuathung [3,6], Tinnakorn Theansungnoen [4], Kanika Thongkhao [5], and Aekkhaluck Intharuksa [1,*]

Supplementary materials

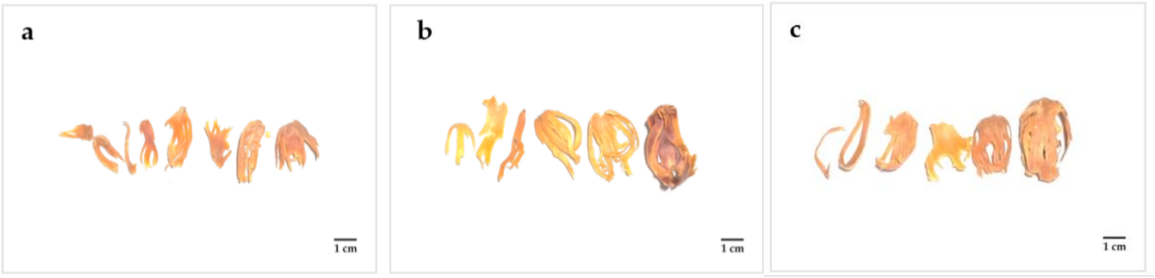

Figure S1 The arils of authentic nutmeg samples: AA1 (**a**), AA2 (**b**), and AA3 (**c**)

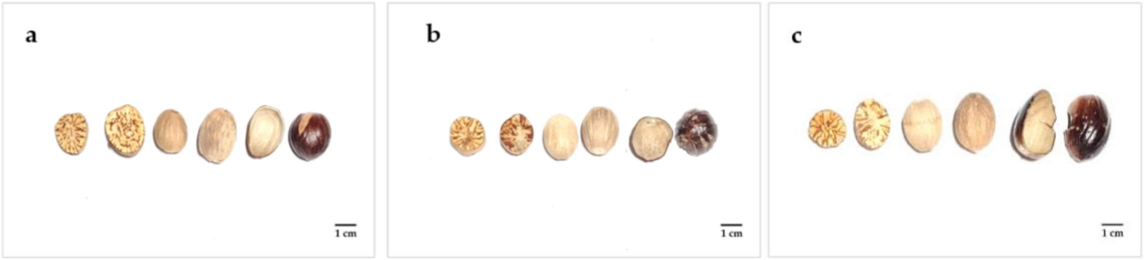

Figure S2 The seeds of authentic nutmeg samples: AS1 (**a**), AS2 (**b**), and AS3 (**c**)

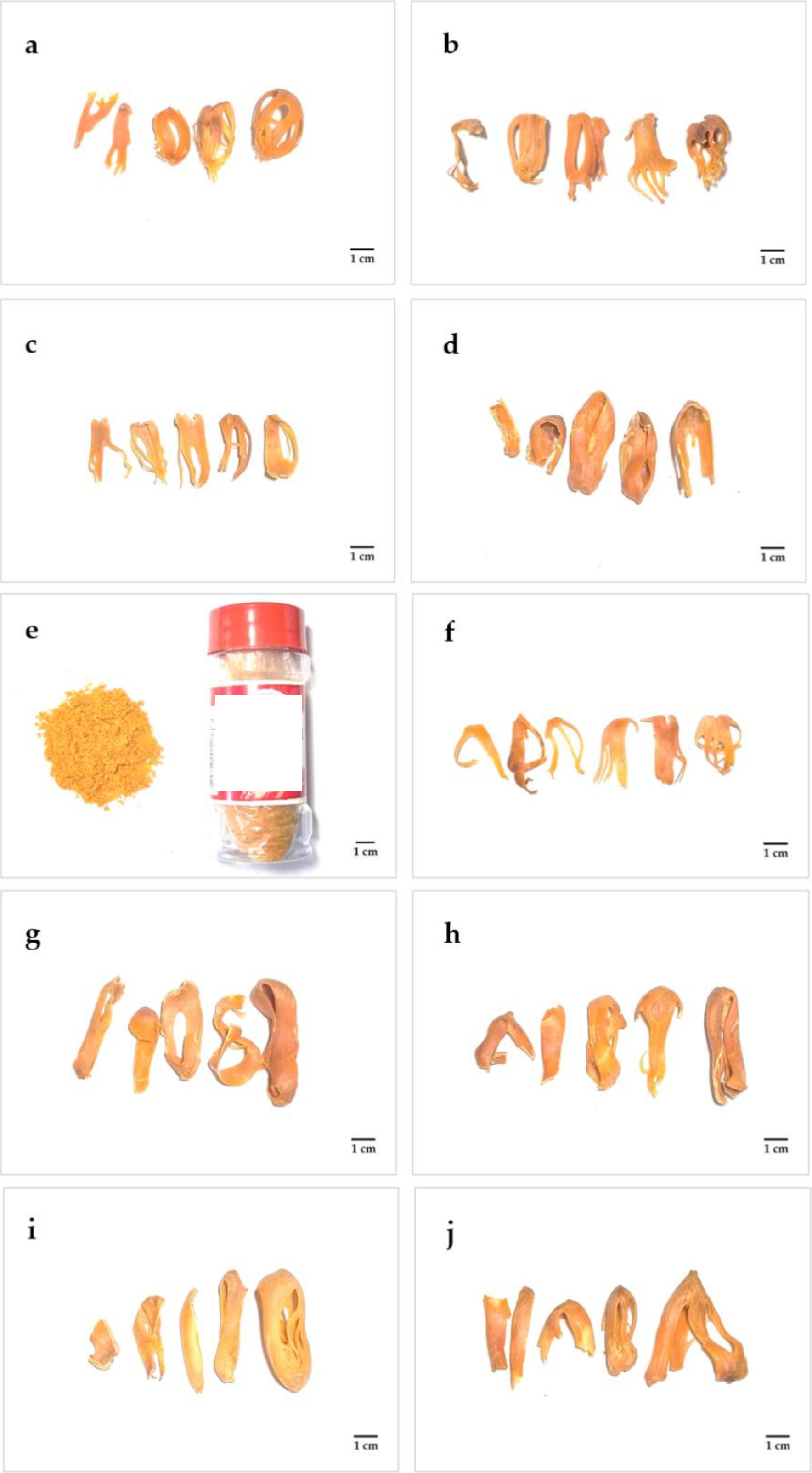

Figure S3 The commercial dried arils and aril product of nutmeg: PA1 (**a**), PA2 (**b**), PA3 (**c**), PA4 (**d**), PA5 (**e**), PA6 (**f**), PA7 (**g**), PA8 (**h**), PA9 (**i**), and PA10 (**j**)

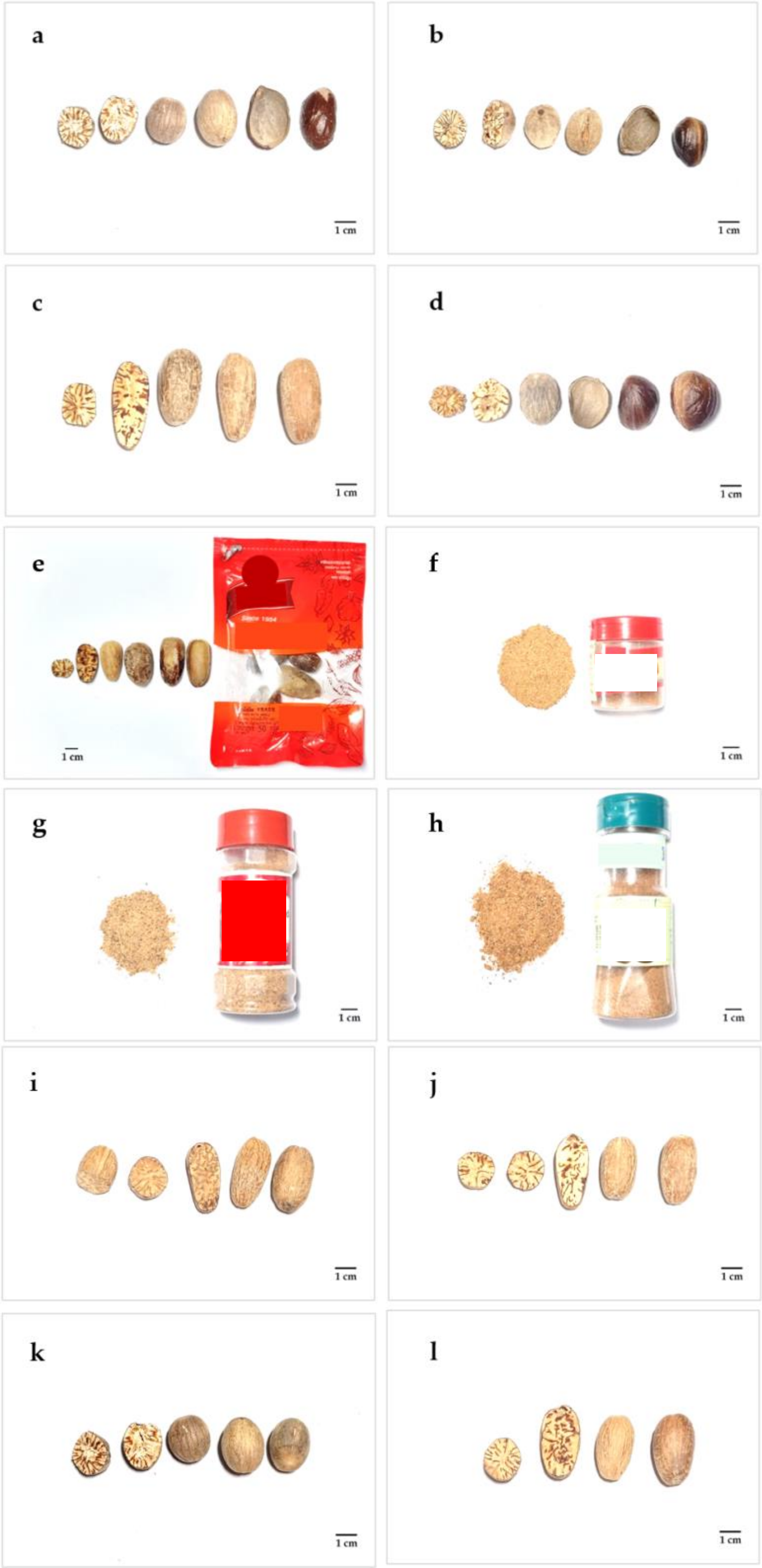

Figure S4 The commercial dried seeds and seed products of nutmeg: PS1 (**a**), PS2 (**b**), PS3 (**c**), PS4 (**d**), PS5 (**e**), PS6 (**f**), PS7 (**g**), PS8 (**h**), PS9 (**i**), PS10 (**j**), PS11 (**k**), and PS12 (**l**)

```
                                ....|....| ....|....| ....|....| ....|....| ....|....|
                                        10         20         30         40         50
M. fragrans                     GGTGCTGTTG AAGCTCTATC AACAAATGGC TAATACTTCG GTATTAGTGT
globose-shaped nutmeg (PS1)     GGTGCTGTTG AAGCTCTATC AACAAATGGC TAATACTTCG GTATTAGTGT
oval-shaped nutmeg (PS3)        GCTGCTGTTG AAGCTCTATC AACAAATGGC TAATACTTCG GTATTAGTGT

                                ....|....| ....|....| ....|....| ....|....| ....|....|
                                        60         70         80         90        100
M. fragrans                     ATACGAGTCA TTGAAGGATC AGATCAATAC CCAGAAGTGG GTATTGATCT
globose-shaped nutmeg (PS1)     ATACGAGTCA TTGAAGGATC AGATCAATAC CCAGAAGTGG GTATTGATCT
oval-shaped nutmeg (PS3)        ATACGAGTCA TTGAAGGATC AGATCAATAC CCACTTCTGG GTATTGATCT

                                ....|....| ....|....| ....|....| ....|....| ....|....|
                                       110        120        130        140        150
M. fragrans                     GGTTCACTAG TGGTTTACTC ACATAAGATT TTTTTTTTTC AATTTCAGAA
globose-shaped nutmeg (PS1)     GGTTCACTAG TGGTTTACTC ACATAAGATT TTTTTTTTTC AATTTCAGAA
oval-shaped nutmeg (PS3)        GGTTCACTAG TGGTTTACTC ACATAAGA-- TTTTTTTTTC AATTTAAGAA

                                ....|....| ....|....| ....|....| ....|....| ....|....|
                                       160        170        180        190        200
M. fragrans                     AATATTAATT ATTTGGTTCA TGATCGAACA TCATACC-TT TTTTTTTTAT
globose-shaped nutmeg (PS1)     AATATTAATT ATTTGGTTCA TGATCGAACA TCATACC-TT TTTTTTTTAT
oval-shaped nutmeg (PS3)        AATATTAATT ATTTGGTTCA TGATCGAACA TCATACCTTT TTTTTTTTAT

                                ....|....| ....|....| ....|....| ....|....| ....|....|
                                       210        220        230        240        250
M. fragrans                     TTTAACTGAT CTTCAGTTTA TGTACATTCC TAAAATGCAT TTTTTCCTAT
globose-shaped nutmeg (PS1)     TTTAACTGAT CTTCAGTTTA TGTACATTCC TAAAATGCAT TTTTTCCTAT
oval-shaped nutmeg (PS3)        TTTAACTGAT CTTCAGTTTA TGTACATTCC TAAAATGAAT TTTTTCCTAT

                                ....|....| ....|....| ....|....| ....|....| ....|....|
                                       260        270        280        290        300
M. fragrans                     TGTCAATTTT ATAAAGGGTA TAAATTAGTA TGAAAAAAAA AAAGAAAGAC
globose-shaped nutmeg (PS1)     TGTCAATTTT ATAAAGGGTA TAAATTAGTA TGAAAAAAAA AAAGAAAGAC
oval-shaped nutmeg (PS3)        TGTCAATTTT ATAAAGGGTA TAAATTAGTA TG-AAAAAAA AAAGAAAGAC

                                ....|....| ....|....| ....|....| ....|....| ....|....|
                                       310        320        330        340        350
M. fragrans                     TAGTAATCTA AAATTTGTGT CTTTAGAAAA GACAAGGGGC GGATGTACCC
globose-shaped nutmeg (PS1)     TAGTAATCTA AAATTTGTGT CTTTAGAAAA GACAAGGGGC GGATGTACCC
oval-shaped nutmeg (PS3)        TAGTAATCTA AAATTTGTGT CTTTAGAAAA GACAAGGGGC GGATGTAGCC

                                ....|....| ..
                                       360
M. fragrans                     AAGGGGATCA AG
globose-shaped nutmeg (PS1)     AAGGGGATCA AG
oval-shaped nutmeg (PS3)        AAGTGGATCA AG
```

Figure S5 Alignment of *trn*H-*psb*A region of authentic *M. fragrans*, commercial globose-shaped nutmeg (PS1), and oval-shaped nutmeg (PS3).

Table S1 Morphological characteristics and size measurements of arils and seeds of authentic *M. fragrans* and commercial nutmeg samples

Specimen Code	Sample Typologies	Color	Odor	Dimensions (cm)		
				Length	Width	Thickness
**Arils**						
Authentic samples						
AA1	Dried arils	Orange	pungent	2.60 ± 0.53	0.90 ± 0.51	0.05 ± 0.01
AA2	Dried arils	Yellowish orange	pungent	3.23 ± 0.60	0.75 ± 0.40	0.06 ± 0.01
AA3	Dried arils	Orange	pungent	3.10 ± 0.53	1.23 ± 0.76	0.04 ± 0.01
Commercial samples						
PA1	Dried arils	Yellowish orange	pungent	2.73 ± 0.75	0.70 ± 0.44	0.04 ± 0.01
PA2	Dried arils	Yellowish orange	pungent	2.77 ± 0.68	1.37 ± 0.68	0.04 ± 0.01
PA3	Dried arils	Yellowish orange	pungent	2.43 ± 0.20	0.88 ± 0.49	0.05 ± 0.01
PA4	Dried arils	Yellowish orange	pungent	3.95 ± 1.18	1.28 ± 0.19	0.06 ± 0.00
PA5	Aril Powder	Yellowish orange	pungent	-	-	-
PA6	Dried arils	Yellowish orange	pungent	2.63 ± 0.51	0.98 ± 0.49	0.05 ± 0.01
PA7	Dried arils	Orange	pungent	3.45 ± 0.88	1.03 ± 0.38	0.06 ± 0.01
PA8	Dried arils	Yellowish orange	pungent	3.68 ± 0.36	0.93 ± 0.23	0.06 ± 0.01
PA9	Dried arils	Yellowish orange	pungent	4.80 ± 0.18	1.00 ± 0.46	0.06 ± 0.01
PA10	Dried arils	Orange	pungent	3.13 ± 0.55	0.87 ± 0.55	0.05 ± 0.00
**Seeds**						
Authentic samples						
AS1	Dried seeds	bright brown	pungent	1.78 ± 0.48	1.40 ± 0.10	-
AS2	Dried seeds	bright brown	pungent	2.00 ± 0.30	1.45 ± 0.05	-
AS3	Dried seeds	bright brown	pungent	2.25 ± 0.22	1.57 ± 0.06	-
Commercial samples						
PS1	Dried seeds	bright brown	pungent	1.90 ± 0.37	1.57 ± 0.06	-
PS2	Dried seeds	bright brown	pungent	2.05 ± 0.57	1.60 ± 0.10	-
PS3	Dried seeds	brown	pungent	3.78 ± 0.19	1.78 ± 0.14	-
PS4	Dried seeds	bright brown	pungent	2.03 ± 0.25	1.47 ± 0.03	-
PS5	Dried seeds	brown	pungent	3.07 ± 0.25	1.60 ± 0.10	-
PS6	Seed Powders	bright brown	pungent	-	-	-
PS7	Seed Powders	brown	pungent	-	-	-
PS8	Seed Powders	bright brown	pungent	-	-	-
PS9	Dried seeds	brown	pungent	2.38 ± 0.28	1.65 ± 0.05	-
PS10	Dried seeds	brown	pungent	3.03 ± 0.25	1.57 ± 0.15	-
PS11	Dried seeds	brown	pungent	2.20 ± 0.10	1.23 ± 0.06	-
PS12	Dried seeds	brown	pungent	2.92 ± 0.14	1.65 ± 0.05	-

Table S2 Primers used in this study

Regions	Primers	Sequence (5′ > 3′)	Reference
ITS	ITS1	CCT TAT CAT TTA GAG GAA GGA G	[1]
	ITS4	TCC TCC GCT TAT TGA TAT GC	[2]
*mat*K	*mat*K-1RKIM-f	ACC CAG TCC ATC TGG AAA TCT TGG TTC	[3]
	*mat*K-3FKIM-r	CGT ACA GTA CTT TTG TGT TTA CGA G	[3]
*rbc*L	*rbc*L1F	ATG TCA CCA CAA ACA GAG ACT AAA GC	[4]
	*rbc*L724R	GTA AAA TCA AGT CCA CCG CG	[4]
*trn*H-*psb*A	*trnH* (GUG)	CGT AAC AAG GTT TCC GTA GGT GAA	[5]
	psbA	GTT ATG CAT GAA CGT AAT GCTC	[5]
*trn*L-F	c	CGA AAT CGG TAG ACG CTA CG	[6]
	f	ATT TGA ACT GGT GAC ACG AG	[6]

Reference:

1. Stanford, A.M.; Harden, R.; Parks, C.R. Phylogeny and biogeography of Juglans (Juglandaceae) based on *mat*K and ITS sequence data. *American Journal of Botany* **2000**, *87*, 872-882.
2. White, T.J.; Bruns, T.; Lee, S.; Taylor, J. Amplification and direct sequencing of fungal ribosomal RNA genes for phylogenetics. *PCR protocols: a guide to methods and applications* **1990**, *18*, 315-322.
3. MB, H. Four primer pairs for the amplification of chloroplast intergenic regions with intraspecific variation. *Mol Ecol* **1999**, *8*, 521-522.
4. Fay, M.F.; Bayer, C.; Alverson, W.S.; de Bruijn, A.Y.; Chase, M.W. Plastid *rbc*L sequence data indicate a close affinity between Diegodendron and Bixa. *Taxon* **1998**, *47*, 43-50.
5. Kuzmina, M.L.; Johnson, K.L.; Barron, H.R.; Hebert, P.D. Identification of the vascular plants of Churchill, Manitoba, using a DNA barcode library. *BMC ecology* **2012**, *12*, 1-11.
6. Taberlet, P.; Gielly, L.; Pautou, G.; Bouvet, J. Universal primers for amplification of three non-coding regions of chloroplast DNA. *Plant molecular biology* **1991**, *17*, 1105-1109.

Table S3 The PCR conditions for DNA amplification of each DNA barcoding regions

**PCR conditions**	**DNA barcoding regions**				
	ITS	*mat*K	*rbc*L	*trn*H-*psb*A	*trn*L-F
Pre-heating	94°C, 2 min	94°C, 2 min	94°C, 2 min	94°C, 2 min	94°C, 2 min
Denaturation	94°C, 15 s	94°C, 15 s	94°C, 15 s	94°C, 15 s	94°C, 15 s
Annealing	54°C, 15 s	52°C, 15 s	52°C, 15 s	56°C, 15 s	52°C, 15 s
Extension	68ºC, 45 s	68ºC, 45 s	68°C, 45 s	68°C, 45 s	68°C, 45 s
Final extension	68°C, 20 min	68°C, 10 min	68°C, 10 min	68°C, 20 min	68°C, 10 min
Cycle (loops)	40	40	35	40	35

Table S4 %AUC of the major phytochemicals found in the volatile oil extracted from the representative aril and seed of globose-shaped and oval-shaped nutmegs

No.	RT	components	%Area Under the Curve (%AUC)			
			AA1	AS1	PA4	PS3
1	6.7	pinene, and terpinen-4-ol	15.68	11.74	3.04	3.41
2	7.9	β-phellandrene	33.94	30.98	18.99	27.00
3	9.7	(5R)-1-methyl-5-(1-methyl ethenyl)cyclohexene	7.43	5.89	20.29	18.71
4		terpinene	3.64	2.91	2.81	2.72
5	15.7	terpinen-4-ol	6.48	4.22	4.34	6.93
6	20.4	safrole	0.32	6.17	33.35	19.61
7	30.1	myristicin	5.23	3.39	0.04	1.48
